# Sodium-glucose cotransporter-2 inhibitors in cancer patients with type 2 diabetes and established immune checkpoint inhibitor-related cardiotoxicity: a retrospective analysis

**DOI:** 10.3389/fendo.2026.1776717

**Published:** 2026-04-15

**Authors:** Ling Guo, Jing Liu, Ruipu Gao, Chunwang Yang, Zhenli Li, Zhengkun Guan, Yansong Wang, Guang Liu, Jingtao Ma, Guangbin Gao

**Affiliations:** 1 Department of Cardiology, The Fourth Hospital of Hebei Medical University, Shijiazhuang, China; 2 Department of Cardiology, Chinese People’s Liberation Army 82nd Army Group Hospital, Baoding, China; 3 Department of immuno-oncology, The Fourth Hospital of Hebei Medical University, Shijiazhuang, China; 4 Department of Radiation Oncology, The Fourth Hospital of Hebei Medical University, Shijiazhuang, China

**Keywords:** all-cause mortality, cancer, iRCs, SGLT2i, T2DM

## Abstract

**Background:**

Immune checkpoint inhibitors (ICIs) significantly improve cancer prognosis but are associated with the risk of immune checkpoint inhibitor-related cardiotoxicity (iRCs), a life-threatening complication. Type 2 Diabetes Mellitus (T2DM) further increases the risk of iRCs and worsens outcomes in these patients. Although sodium-glucose cotransporter-2 inhibitors (SGLT2i) confer cardioprotective and potential antitumor effects, their prognostic value in cancer patients with T2DM and established iRCs remains unknown.

**Objective:**

To investigate the association of SGLT2i use with all-cause mortality, iRCs severity, and major adverse cardiovascular events (MACE) in cancer patients with T2DM who developed iRCs during ICI therapy.

**Methods:**

In this retrospective study, we analyzed 98 cancer patients with T2DM and established iRCs between January 2019 and June 2025. Participants were categorized into an SGLT2i group (n = 26) and a non-SGLT2i group (n = 72). The primary endpoint was all-cause mortality; secondary endpoints included 40-day MACE and iRCs severity. Survival analyses were performed using Kaplan-Meier curves with the log-rank test. Independent associations were assessed via Cox proportional hazards regression.

**Results:**

Median follow-up was 950.5 days. SGLT2i use was independently associated with reduced all-cause mortality (adjusted HR = 0.520, 95% CI: 0.285–0.947, p = 0.033). The SGLT2i group exhibited a longer median survival time (743 days vs. 494 days) and consistently higher 1-, 2-, and 3-year survival rates (73.1% vs. 60.6%; 51.5% vs. 26.4%; 31.2% vs. 8.8%) compared to the non-SGLT2i group. Additionally, the SGLT2i group had a significantly lower proportion of high-grade iRCs (19.2% vs. 45.8%, p = 0.031). Although the incidence of MACE did not differ significantly between groups (19.2% vs. 33.3%, p = 0.271), univariate Cox regression indicated a 47% lower risk of MACE in the SGLT2i group (HR = 0.531, 95% CI: 0.202–1.391, p = 0.198), with numerical reductions observed for both overall MACE and its individual components.

**Conclusion:**

SGLT2i use in cancer patients with T2DM and established iRCs was independently associated with lower all-cause mortality and linked to a reduced incidence of high-grade iRCs and favorable MACE trends. These findings warrant prospective validation to confirm cardioprotective and potential oncologic benefits of SGLT2i in this high-risk population.

## Introduction

ICIs have transformed the therapeutic landscape for malignant tumors, offering the possibility of long-term survival to many patients with solid cancers (1, 2). However, while activating the body’s anti-tumor immune response, ICIs may trigger immune-related adverse events (irAEs) (3). Among these, ICI-related cardiotoxicity (iRCs) has an incidence of only 1–5%, but its insidious onset, rapid progression, and high mortality make it a critical bottleneck limiting the safe use of ICIs (4–6). The pathological mechanism of iRCs centers on immune infiltration of myocardial tissue and cytokine storms, with clinical manifestations including myocarditis, severe arrhythmias, and heart failure. Currently, clinical management mainly relies on acute-phase immunosuppressive therapies such as glucocorticoids and lacks targeted strategies to improve long-term prognosis (7, 8).

T2DM is a prevalent comorbidity in cancer patients. Epidemiological evidence indicates that T2DM not only elevates the risk of cancer incidence and mortality (9–11) but may also impair the efficacy of immunotherapy (12). Notably, T2DM has been shown to augment the risk of iRCs, aggravate the severity of cardiac injury, and contribute to poorer outcomes in affected patients (13). While the 2022 European Society of Cardiology (ESC) cardio-oncology guidelines establish diagnostic criteria for iRCs, they offer no specific recommendations regarding the choice of glucose-lowering agents for patients with concomitant T2DM (8). Consequently, a critical gap remains in the development of integrated clinical strategies that effectively balance glycemic control, cardioprotection, and oncologic therapy.

SGLT2i are a class of glucose-lowering agents known to confer multidimensional benefits extending beyond glycemic control. Substantial clinical evidence has demonstrated that SGLT2i significantly reduces the incidence of MACE in patients with T2DM, mediated through mechanisms including anti-inflammatory effects, oxidative stress inhibition, and improved mitochondrial function (14–16). Concurrently, accumulating evidence points to potential antitumor properties of SGLT2i. Proposed mechanisms include the inhibition of tumor cell glucose uptake (17), reversal of the Warburg effect (18, 19), suppression of the AKT/mTOR proliferation pathway, activation of the AMPK pro-apoptotic pathway (20), and modulation of tumor cell-derived chemokines and cytokines to remodel the tumor microenvironment (21). These actions may collectively inhibit tumor progression and provide additional survival benefits to cancer patients (22). A recent retrospective study by Perelman et al. further reported that SGLT2i use was associated with reduced all-cause mortality in patients with T2DM receiving ICIs for cancer (23). However, that study encompassed a broad ICI-treated population and did not specifically examine the high-risk subgroup with established iRCs.

Given the dismal prognosis and the absence of targeted therapies for cancer patients with concurrent T2DM and iRCs, coupled with the established cardioprotective and potential antitumor properties of SGLT2i, this study aimed to evaluate the effect of SGLT2i use, initiated prior to iRCs onset, on all-cause mortality, MACE, and iRCs severity, thus clarifying its role in the overall prognosis of this high-risk cohort. To our knowledge, this represents the first investigation specifically targeting cancer patients with T2DM and established iRCs. Our findings are anticipated to provide critical evidence to address the current therapeutic gap and inform optimized management strategies for this vulnerable population.

## Materials and methods

### Study design and population

This single-center, retrospective analysis was conducted at the Fourth Hospital of Hebei Medical University (Shijiazhuang, China). We screened and enrolled 98 consecutive patients with cancer and T2DM who developed iRCs during ICI treatment between January 2019 and June 2025.

Inclusion criteria were: (1) age ≥18 years; (2) diagnosis of both cancer and T2DM; (3) treatment with at least one cycle of ICI (as monotherapy or combination therapy); (4) iRCs diagnosis conforming to the 2022 ESC Cardio-Oncology Guidelines and IC-OS Consensus Statement (7, 8); and (5) final confirmation of iRCs by a senior cardiologist and oncologist.

Exclusion criteria were: (1) insufficient medical records for endpoint adjudication; and (2) receipt of other antitumor therapies without concurrent ICI treatment.

Eligible patients were categorized into two groups: the SGLT2i group (patients on SGLT2i treatment prior to iRCs diagnosis) and the non-SGLT2i group (patients without SGLT2i treatment).

### Data collection

Comprehensive patient data were collected, including demographics, smoking history, comorbidities, tumor characteristics, prior and current antitumor therapies, ICI regimen details, iRCs event profiles, standard laboratory results, and echocardiographic parameters. All laboratory measurements were performed in the central laboratory of the Fourth Hospital of Hebei Medical University using standardized protocols.

### Definition of iRCs

ICI-related cardiotoxicity encompasses a broad spectrum of cardiac manifestations, which can be categorized as inflammatory or non-inflammatory. Inflammatory cardiotoxicity includes myocarditis, perimyocarditis, pericarditis, and isolated left ventricular dysfunction. Non-inflammatory cardiotoxicity comprises asymptomatic left ventricular dysfunction, takotsubo-like syndrome, coronary vasospasm, arrhythmias, and myocardial infarction (24, 25).

### Follow-up and outcome

The primary endpoint was all-cause mortality, measured from the date of iRCs diagnosis to death from any cause.

Secondary endpoints included: (1) iRCs severity: Graded at the time of iRCs diagnosis using the Common Terminology Criteria for Adverse Events (CTCAE) v5.0. Events were classified as low-grade (CTCAE ≤2) or high-grade (CTCAE ≥3). (2) MACE: Assessed within 40 days after iRCs diagnosis. MACE was a composite of cardiovascular death, cardiac arrest, cardiogenic shock, and hemodynamically significant complete heart block, as defined in prior studies (26, 27).

Patients were monitored for both short-term (40-day) and long-term outcomes. iRCs severity was determined at diagnosis via clinical, laboratory, and imaging evaluation. Data on MACE and all-cause mortality were collected through inpatient monitoring and structured telephone follow-ups.

### Statistical analysis

Continuous variables are expressed as mean ± standard deviation (SD) for normally distributed data or median [interquartile range (IQR)] for non-normally distributed data. Categorical variables are summarized as frequencies (percentages). Between-group comparisons were performed using the two-tailed unpaired t-test (normal distribution) or Mann-Whitney U test (non-normal distribution) for continuous variables, and the Chi-square or Fisher’s exact test for categorical variables.

The median follow-up duration was calculated using the direct method, with individual follow-up defined as the time from the iRCs diagnosis to the last follow-up. Survival outcomes were visualized with Kaplan-Meier curves and compared using the log-rank test (overall survival for all-cause mortality; MACE-free survival for 40-day MACE). The association between SGLT2i use and all-cause mortality was quantified using univariate and multivariable Cox proportional hazards models, reported as HR with 95% CI; the multivariable model was adjusted for age, gender, cancer type/stage, treatment protocol, and cardiovascular risk factors. For the 40-day MACE endpoint, only univariate Cox regression was applied. A two-sided p-value < 0.05 was considered statistically significant. All analyses were conducted using R software version 4.5.2 (R Foundation for Statistical Computing, Vienna, Austria).

## Results

### Baseline patient characteristics

The final cohort comprised 98 patients with cancer and T2DM who were diagnosed with iRCs. Of these, 26 (26.5%) were classified into the SGLT2i group and 72 (73.5%) into the non-SGLT2i group. Baseline characteristics of the entire cohort and comparisons between groups are summarized in **Table 1**.

**Table 1:** Characteristics of enrolled patients.

Characteristics	All participants N=98	Non-SGLT2i N=72	SGLT2i N=26	P value
Basic information				
Age, years	67 [62.25,71]	67 [60.75,71]	67.5 [63.25,75.5]	0.530
Males, n (%)	73(74.5)	56 (77.8)	17 (65.4)	0.327
ECOG PS	1.0 [1.0, 3.0]	1.0 [1.0, 3.0]	1.5 [1.0, 2.0]	0.804
BMI, kg/m^2^	22.87 ± 3.33	22.63 ± 3.29	23.53 ± 3.40	0.238
T2DM duration, years	8.00 [5.00, 13.00]	8.50 [5.00, 13.00]	7.00 [4.00, 12.75]	0.420
Smoking, n (%)	42 (42.9)	32 (44.4)	10 (38.5)	0.766
Comorbid Conditions				
hypertension, n (%)	39 (39.8)	29 (40.3)	10 (38.5)	1.000
Hyperlipidemia, n (%)	30 (30.6)	18 (25.0)	12 (46.2)	0.079
Previous CVD, n (%)	37 (37.8)	28 (38.9)	9 (34.6)	0.881
History of Stroke, n (%)	12 (12.2)	6 (8.3)	6 (23.1)	0.106
Tumor Characteristics				
Cancer Type				0.724
NSCLC	30 (30.6)	22 (30.6)	8 (30.8)	
ESCC	23 (23.5)	15 (20.8)	8 (30.8)	
GC	20 (20.4)	16 (22.2)	4 (15.4)	
Other	25 (25.5)	19 (26.4)	6 (23.1)	
Cancer stage, n (%)				0.257
II	3 (3.1)	1 (1.4)	2 (7.7)	
II	31 (31.6)	24 (33.3)	7 (26.9)	
IV	64 (65.3)	47 (65.3)	17 (65.4)	
Prior Antitumor Therapy				
Surgery, n (%)	15 (15.3)	9 (12.5)	6 (23.1)	0.334
Radiotherapy, n (%)	21 (21.4)	16 (22.2)	5 (19.2)	0.968
Chemotherapy, n (%)	78 (79.6)	56 (77.8)	22 (84.6)	0.647
Targeted Therapy, n (%)	24 (24.5)	18 (25.0)	6 (23.1)	1.000
ICI Treatment Details				
ICI Type				0.673
PD-1, n (%)	87 (88.8)	65 (90.3)	22 (84.6)	
PD-L1, n (%)	11 (11.2)	7 (9.7)	4 (15.4)	
ICI Cycles	2.0 [1.0, 4.0]	2.0 [1.0, 4.0]	2.0 [1.0, 3.0]	0.474
iRCs Presentation				
iRCs Type				0.256
Myocarditis, n (%)	52 (53.1)	39 (54.2)	13 (50.0)	
Arrhythmias, n (%)	24 (24.5)	19 (26.4)	5 (19.2)	
Impaired ventricular function, n (%)	18 (18.4)	12 (16.7)	6 (23.1)	
Acute myocardial infarction, n (%)	2 (2.0)	2 (2.8)	0 (0.0)	
Takotsubo-like syndrome, n (%)	1 (1.0)	0 (0.0)	1 (3.8)	
Pericardial diseases, n (%)	1 (1.0)	0 (0.0)	1 (3.8)	
Days to iRCs	55.00 [31.00, 97.25]	56.50 [31.00, 98.25]	49.00 [30.75, 71.00]	0.584
Concomitant non-cardiac irAEs, n (%)	33 (33.7)	23 (31.9)	10 (38.5)	0.718
Laboratory Parameters				
LDH, U/L	319.50 [219.75, 645.50]	345.50 [237.25, 640.50]	304.50 [206.25, 637.50]	0.557
CKMB, ng/ml	30.80 [10.35, 86.25]	31.60 [11.10, 101.90]	25.50 [9.88, 53.35]	0.509
Myoglobin, ng/ml	67.00 [43.38, 302.85]	60.90 [41.52, 298.55]	76.10 [55.47, 344.55]	0.541
cTnI, ng/ml	0.08 [0.01, 0.34]	0.10 [0.02, 0.35]	0.06 [0.01, 0.24]	0.186
NT-proBNP, pg/ml	416.00 [129.00, 2062.50]	278.00 [124.50, 1845.00]	594.00 [149.00, 3120.00]	0.214
Echocardiography				
LVEF, %	61.00 [56.00, 64.00]	60.00 [56.00, 63.25]	63.00 [57.25, 65.00]	0.345

Data are presented as n (%), mean ± standard deviation, or median [interquartile range]. SGLT2i, Sodium-glucose Cotransporter 2 Inhibitors; ECOG PS, Eastern Cooperative Oncology Group Performance Status; BMI, Body Mass Index; CVD, cardiovascular disease; NSCLC, Non-Small Cell Lung Cancer, ESCC, Esophageal Squamous Cell Carcinoma; GC, Gastric cancer; ICI, Immune Checkpoint Inhibitor; PD-1, Programmed Death 1; PD-L1, Programmed Death Ligand 1; iRCs, Immune Checkpoint Inhibitor-related Cardiotoxicity; irAEs, Immune-related Adverse Events; LDH, Lactate Dehydrogenase; CKMB, Creatine Kinase-Myocardial Band; Myo, Myoglobin; cTnI, Cardiac Troponin I; NT-proBNP, N-terminal Pro-B-type Natriuretic Peptide; LVEF, Left Ventricular Ejection Fraction.

The overall cohort had a median age of 67 years and was predominantly male (74.5%). The two groups were well-balanced, with no significant differences in demographics, cardiovascular risk factors, tumor characteristics, ICI treatment details, key laboratory parameters, or left ventricular ejection fraction (LVEF). The most common malignancies were non-small cell lung cancer (NSCLC, 30.6%), esophageal squamous cell carcinoma (ESCC, 23.5%), and gastric cancer (GC, 20.4%). The “Other” category (25.5%) encompassed a diverse range of less frequent tumor types, the full spectrum of which is detailed in **Supplementary Table S1**.

### Primary endpoint

**Figure 1:**
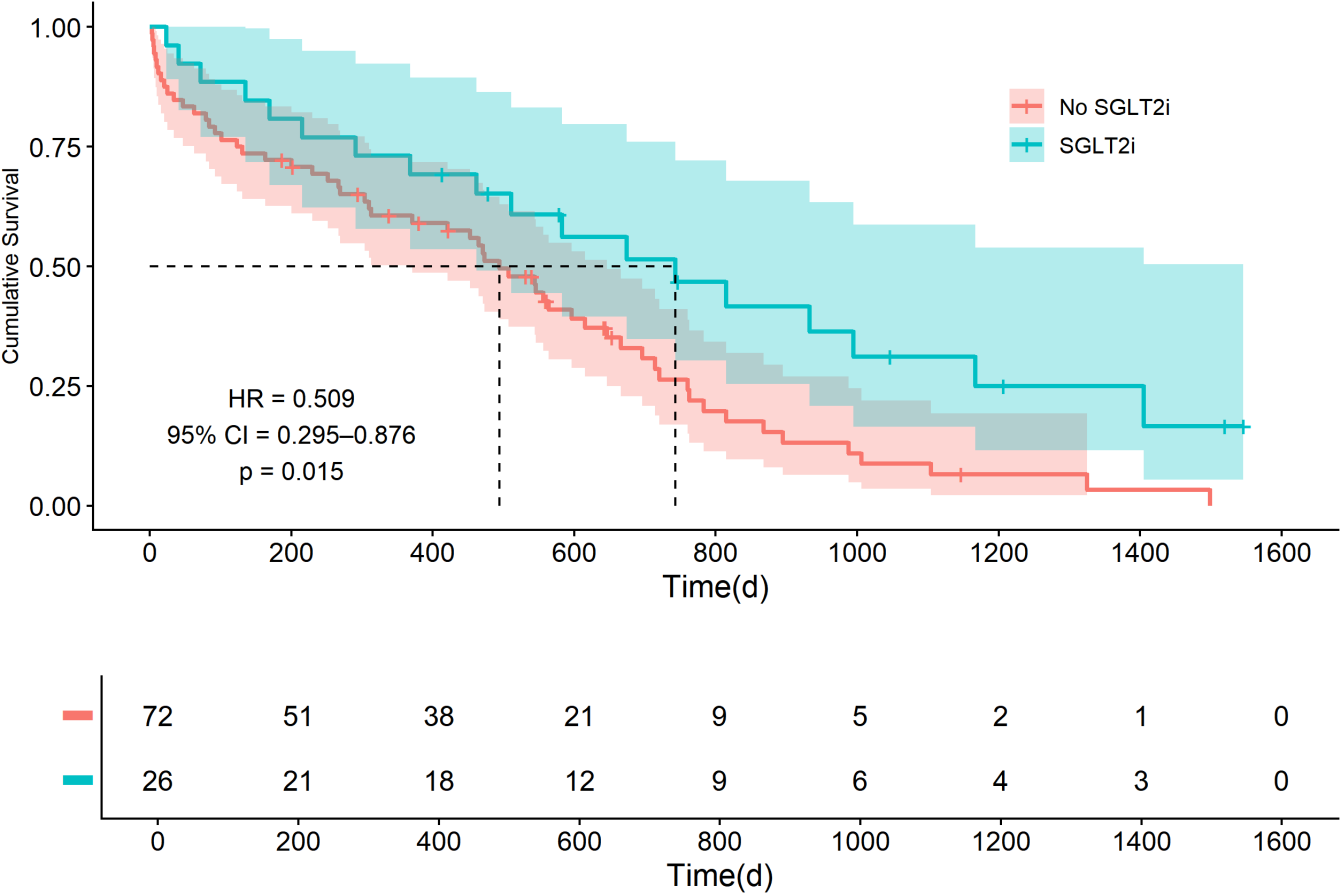
Kaplan-Meier curves for all-cause mortality. The hazard ratio (HR), 95% confidence interval (CI), and p-value are derived from a univariate Cox proportional hazards regression. The corresponding log-rank p-value was 0.013. SGLT2i, sodium-glucose cotransporter-2 inhibitors.

Over a median follow-up of 950.5 days (IQR: 561.5–1460.2), all-cause mortality occurred in 76 patients (77.6%). The Kaplan-Meier survival analysis demonstrated a significant separation of the survival curves, favoring the SGLT2i group. This visual finding was quantified by univariate Cox proportional hazards regression, which showed that SGLT2i use was associated with a 49% lower risk of all-cause mortality (HR = 0.509, 95% CI: 0.295–0.876, p = 0.015) (**Figure 1**).

The clinical benefit associated with SGLT2i was further evidenced by a substantially longer median survival time (743 days, 95% CI: 462–NA vs. 494 days, 95% CI: 371–646) and consistently higher survival rates at 1, 2, and 3 years (73.1% vs. 60.6%; 51.5% vs. 26.4%; and 31.2% vs. 8.8%, respectively).

After multivariable adjustment for age, gender, cancer type, stage, and cardiovascular risk factors, SGLT2i use remained independently associated with a significantly lower risk of death (adjusted HR = 0.520, 95% CI: 0.285–0.947, p = 0.033), as detailed in **Table 2**.

**Table 2:** Multivariable cox proportional hazards regression analysis for all-cause mortality.

Variable	Adjusted HR	95% CI		P value
		Lower	Upper	
**SGLT2i**	0.520	0.285	0.947	0.033*
Age	0.987	0.964	1.010	0.261
Male	1.520	0.847	2.732	0.161
Cancer type				0.265
NSCLC †	1.250	0.665	2.337	0.492
ESCC †	0.714	0.341	1.496	0.372
GC †	1.500	0.736	3.065	0.264
Stage IV ‡	1.610	0.882	2.930	0.121
Hyperlipidemia	1.170	0.649	2.119	0.597
Hypertension	1.180	0.697	2.009	0.532
Previous CVD	1.270	0.763	2.124	0.355
Stroke	1.940	0.905	4.159	0.088

The Cox proportional hazards regression model was adjusted for all variables listed in the table. *P < 0.05, considered statistically significant.

†The reference category for Cancer type is Other cancers.

‡The reference category for cancer staging is combined Stages II and III.

HR, hazard ratio; CI, confidence interval; SGLT2i, sodium-glucose cotransporter-2 inhibitors; NSCLC, non-small cell lung cancer; ESCC, esophageal squamous cell carcinoma; GC, gastric cancer; CVD, cardiovascular disease.

### Secondary endpoints

**Figure 2:**
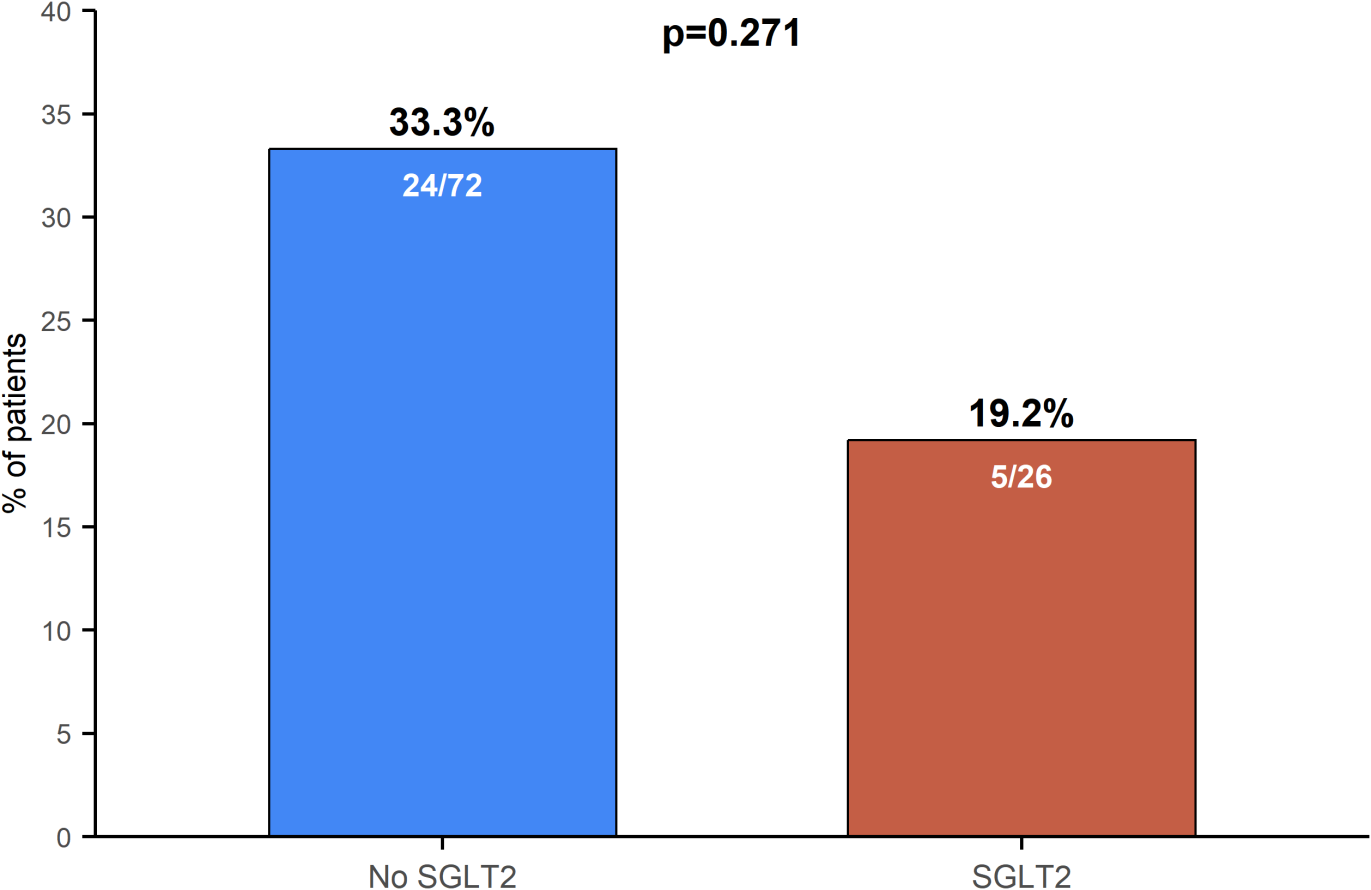
Comparison of MACE incidence between study groups (p=0.271, Chi-square test). MACE, major adverse cardiovascular events; SGLT2i, Sodium-glucose Cotransporter 2 Inhibitors.

The incidence and timing of secondary endpoints are detailed in **Table 3**. A total of 29 patients (29.6%) experienced a MACE within the 40-day follow-up period, with a median time to onset of 4.0 days (IQR: 2.0–8.0) after iRCs diagnosis. The proportion of patients with MACE was numerically lower in the SGLT2i group (5/26, 19.2%) compared to the non-SGLT2i group (24/72, 33.3%), although this difference was not statistically significant (p=0.271; **Figure 2**). This trend toward risk reduction with SGLT2i use was consistent across all individual components of the MACE composite endpoint, including cardiovascular death, cardiac arrest, cardiogenic shock, and hemodynamically significant complete heart block (HS-CHB), with no cardiovascular deaths occurring in the SGLT2i group (**Table 3**).

**Table 3:** Primary and secondary endpoints.

Endpoint	Entire Cohort	Non-SGLT2i	SGLT2i	P value
Primary Endpoint				
All-cause mortality, n (%)	76 (77.6)	58 (80.6)	18 (69.2)	0.362
Secondary Endpoints				
MACEs, n (%)	29 (29.6)	24 (33.3)	5 (19.2)	0.271
Cardiovascular death, n (%)	2 (2.0)	2 (2.8)	0 (0.0)	1.000
Cardiac arrest, n (%)	4 (4.1)	3 (4.2)	1 (3.8)	1.000
Cardiogenic shock, n (%)	12 (12.2)	10 (13.9)	2 (7.7)	0.507
HS-CHB, n (%)	11 (11.2)	9 (12.5)	2 (7.7)	0.722
Days to MACEs†	4.00 [2.00, 8.00]	3.50 [2.00, 8.00]	5.00 [2.00, 6.00]	0.977
Grade of iRCs				0.031*
Low-grade iRCs	60 (61.2)	39 (54.2)	21 (80.8)	
High-grade iRCs	38 (38.8)	33 (45.8)	5 (19.2)	

*P < 0.05, considered statistically significant.

†Presented only for the 29 patients who experienced MACEs.

Non-SGLT2i, Non-Sodium-glucose Cotransporter 2 Inhibitors; SGLT2i, Sodium-glucose Cotransporter 2 Inhibitors; MACE, Major Adverse Cardiovascular Events; HS-CHB, Hemodynamically Significant Complete Heart Block; iRCs, Immune Checkpoint Inhibitor-related Cardiotoxicity.

**Figure 3:**
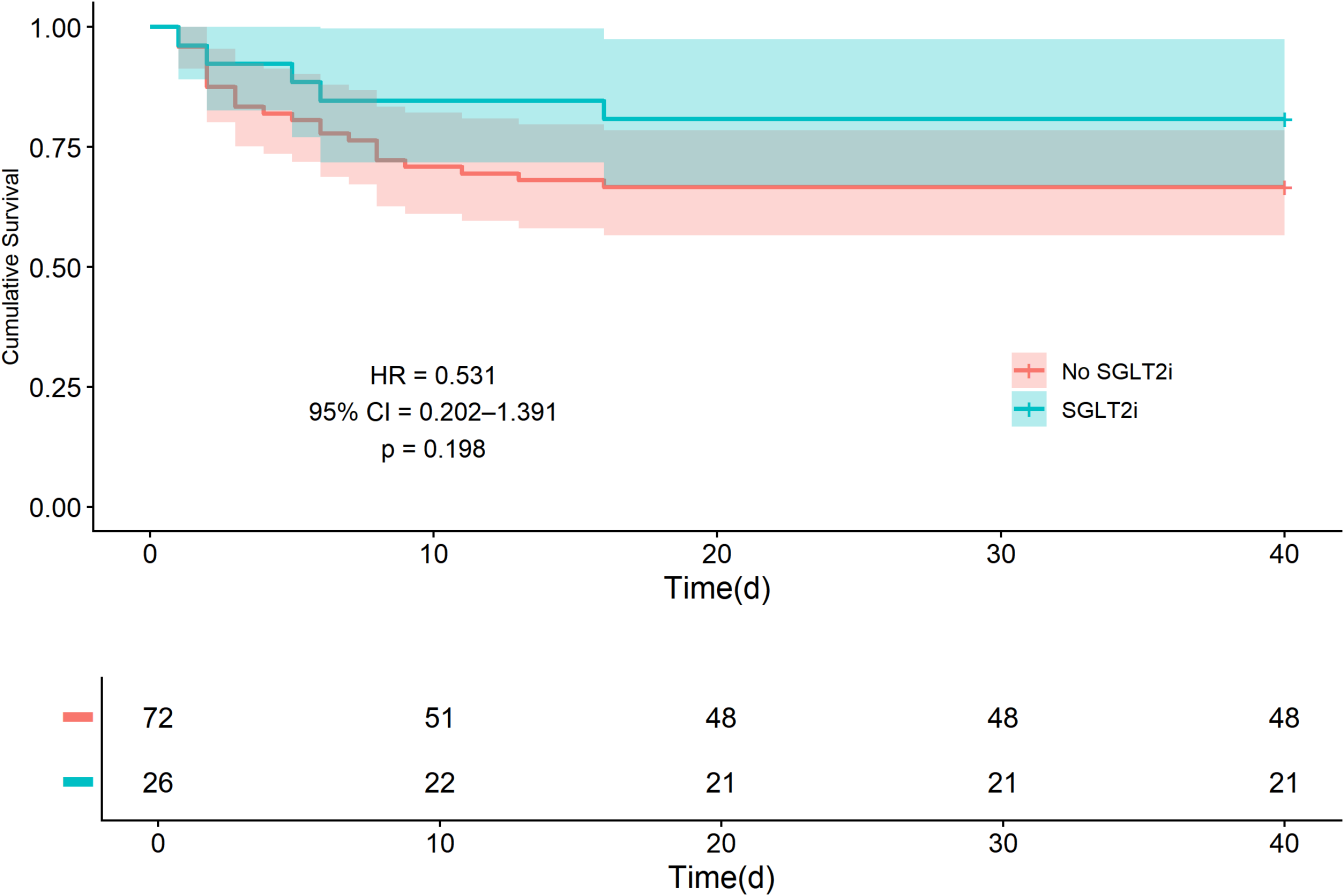
Kaplan-Meier curves for 40-day MACE-free survival. The hazard ratio (HR), 95% confidence interval (CI), and p-value are derived from a univariate Cox proportional hazards regression. The corresponding log-rank p-value was 0.190. MACE, major adverse cardiovascular events; SGLT2i, sodium-glucose cotransporter-2 inhibitors.

The Kaplan-Meier curve for 40-day MACE-free survival showed no significant separation between the groups (log-rank p = 0.190). Quantification using univariate Cox regression was consistent with this visual analysis, indicating a non-significant trend toward benefit with SGLT2i use (HR = 0.531, 95% CI: 0.202–1.391, p = 0.198) (**Figure 3**).

Critically, however, a significant difference was observed in iRCs severity. The proportion of patients with high-grade iRCs (CTCAE ≥3) was substantially lower in the SGLT2i group than in the non-SGLT2i group (19.2% vs. 45.8%, p=0.031).

## Discussion

This study evaluated the prognostic impact of SGLT2i in cancer patients with T2DM who developed iRCs. We observed that SGLT2i use was independently associated with significantly improved survival in this high-risk cohort. To the best of our knowledge, this is the first investigation specifically focusing on this high-risk population, thereby bridging a critical evidence gap regarding clinical management strategies and informing an integrated clinical approach for these complex patients.

In our cohort, SGLT2i use prior to iRCs onset emerged as an independent predictor of reduced all-cause mortality. Multivariable Cox regression analysis demonstrated that, after adjustment for age, sex, cancer type and stage, and cardiovascular risk factors, SGLT2i use remained significantly associated with lower all-cause mortality (HR = 0.520, 95% CI: 0.285–0.947, p=0.033). Compared with the non-SGLT2i group, patients in the SGLT2i group exhibited a 48% reduction in all-cause mortality, a significantly prolonged median survival time (743 vs. 494 days), and consistent advantages in 1-, 2-, and 3-year survival rates (73.1% vs. 60.6%; 51.5% vs. 26.4%; and 31.2% vs. 8.8%, respectively). This long-term survival benefit is particularly noteworthy. Although the incidence of MACE did not differ significantly between groups (19.2% vs. 33.3%, p=0.271), univariate Cox regression suggested a trend toward a 47% lower risk of MACE with SGLT2i use (HR = 0.531, 95% CI: 0.202–1.391, p=0.198). A numerical reduction was observed across all MACE components—including cardiovascular death, cardiac arrest, cardiogenic shock, and hemodynamically significant complete heart block—with no cardiovascular deaths reported in the SGLT2i group. Importantly, at the time of iRCs diagnosis, the proportion of high-grade iRCs was significantly lower in the SGLT2i group (19.2% vs. 45.8%, p=0.031), suggesting that SGLT2i may improve subsequent survival by attenuating iRCs severity.

These findings align with and extend the results of a recent study by Perelman et al. (23), which reported a 70% reduction in all-cause mortality among SGLT2i users in a broader cohort of T2DM cancer patients receiving ICI therapy (HR = 0.300), noting no cases of myocarditis or atrial fibrillation. However, that study did not specifically analyze the high-risk subgroup with established iRCs. As one of the most fatal adverse events during ICI therapy, iRCs herald a marked deterioration in patient prognosis (5). By specifically targeting this high-risk population, our study confirms that the protective effect of SGLT2i persists in such subgroups, thereby strengthening its value in cardio-oncology and providing more targeted evidence for clinical decision-making.

The observed clinical benefits of SGLT2i may stem from their multidimensional synergistic protective mechanisms. First, the core pathology of iRCs involves immune-mediated inflammatory responses and cytokine storms, wherein the infiltration of aberrantly activated T cells and the release of pro-inflammatory factors in myocardial tissue act as key drivers of cardiac injury (28). SGLT2i have been shown to exert potent anti-inflammatory and immunomodulatory effects, inhibiting pro-inflammatory cytokine release and reducing myocardial inflammatory infiltration (29). This mechanism offers a direct and plausible explanation for the observed reduction in iRCs severity. By preemptively modulating immune responses, SGLT2i may prevent the escalation of subclinical cardiac inflammation into fulminant, high-grade iRCs, thereby laying the groundwork for improved long-term survival. Second, SGLT2i can directly benefit cardiomyocytes by improving mitochondrial function and attenuating oxidative stress and metabolic disturbances (14), thereby lowering the risk of severe arrhythmias and heart failure (15, 16). This aligns with the cardiovascular protective effects observed in large-scale trials such as DAPA-HF (30) and EMPEROR-Reduced (31). The lack of statistical significance for MACE reduction in our study is likely attributable to the small sample size of the SGLT2i group (n=26) and the low incidence of MACE components, which limited statistical power. Future multicenter studies with larger sample sizes are warranted to further validate this cardioprotective trend.

Notably, the survival benefit associated with SGLT2i persisted throughout extended follow-up, suggesting that their beneficial effects transcend acute-phase cardioprotection and that their potential antitumor properties may serve as an important complementary mechanism. Accumulating evidence supports the additional benefits of SGLT2i in cancer patients. Gongora et al. (32) reported that among cancer patients with comorbid diabetes receiving anthracycline therapy, SGLT2i users experienced lower all-cause mortality and cardiac event rates. Similarly, Chiang et al. (33) observed that SGLT2i use in cancer patients with comorbid T2DM was associated with reduced heart failure incidence and prolonged survival. Another study confirmed that SGLT2i use was associated with reduced cancer-specific and all-cause mortality in T2DM patients with cancer, demonstrating a dose-dependent effect whereby higher cumulative doses correlated with greater survival improvement (22). The antitumor effects of SGLT2i may be closely linked to their ability to regulate tumor cell biology, particularly in cancers overexpressing SGLT2 transporters (17, 34). Specifically, SGLT2i may inhibit tumor progression through multiple pathways: (1) by directly inhibiting tumor cell glucose uptake (17) and reducing Warburg effect-mediated energy supply (18, 19), thereby disrupting tumor cell energy metabolism; (2) by inhibiting the AKT/mTOR proliferation pathway while activating the AMPK pro-apoptotic pathway (20), thus directly modulating tumor cell proliferation and apoptosis; and (3) by remodeling the tumor microenvironment via the regulation of chemokines and cytokines released by tumor cells (21, 35). The sustained long-term survival benefit observed in the SGLT2i group—without attenuation after the acute phase of iRCs—and the absence of an increased risk of cancer-related death indirectly support the potential contribution of these antitumor mechanisms.

Our findings hold significant implications for clinical practice. Although the 2022 ESC Cardio-Oncology Guidelines (8) emphasize the diagnosis and management of iRCs, they offer no specific recommendations regarding glucose-lowering drug selection for patients with concomitant T2DM. Consequently, an integrated clinical strategy balancing glycemic control, cardioprotection, and cancer therapy remains lacking. Our results suggest that initiating or continuing SGLT2i therapy represents a promising strategy to improve prognosis in high-risk populations—specifically, cancer patients with T2DM who develop iRCs during ICI treatment. Furthermore, SGLT2i and glucocorticoids may play complementary roles in iRCs management: glucocorticoids primarily target excessive immune activation in the acute phase, providing rapid anti-inflammatory effects, whereas SGLT2i may contribute to long-term inflammation regulation, myocardial function preservation, and potential tumor inhibition. The synergistic protective potential of combining these two agents warrants further investigation.

Several limitations of this study must be acknowledged. First, the single-center, retrospective design may introduce selection bias and unmeasured confounding factors, such as glucocorticoid dosage and duration after iRCs onset, and subsequent antitumor treatment regimens. Propensity score matching was not utilized to avoid further sample size attrition, which might otherwise have minimized intergroup differences and enhanced result reliability. Second, the relatively small sample size of the SGLT2i group (n=26) and the low incidence of MACE components likely limited statistical power and precluded meaningful subgroup analyses (e.g., by specific iRC type and cancer type) to further identify the populations deriving the greatest benefit from SGLT2i. Third, the lack of data on SGLT2i dosage and treatment duration precluded analysis of efficacy differences across dosing regimens, an area requiring attention in future research.

Future studies should focus on the following directions: conducting prospective, multicenter studies including all ICI-treated patients with T2DM (both with and without iRCs) to validate the efficacy of SGLT2i, clarify its effect on iRCs incidence, and further confirm its benefits in the high-risk subgroup with established iRCs; incorporating standardized data collection of HbA1c and setting pericardial fat assessment as a key exploratory endpoint to explore its predictive value in iRCs progression and heart failure risk; determining the optimal dosage and timing for SGLT2i initiation; exploring the synergistic protective effects of combined SGLT2i and glucocorticoid therapy in iRCs management; and performing stratified analyses by specific iRC type and cancer type to identify potential differences in SGLT2i benefits across cardiac injury subtypes and tumor histotypes, thereby providing more robust evidence for clinical precision therapy. Additionally, in-depth exploration of the specific molecular mechanisms underlying the protective effects of SGLT2i in cardio-oncology is needed to establish a theoretical foundation for developing more precise therapeutic targets.

## Conclusion

In conclusion, SGLT2i use was associated with significantly improved survival, a markedly lower incidence of severe iRCs, and favorable trends in cardiovascular outcomes. Given the exploratory nature of this single-center, retrospective analysis, our findings warrant validation in large-scale, prospective studies to confirm the benefits of SGLT2i and elucidate their underlying cardioprotective and potential oncologic mechanisms in this high-risk population.

## Data Availability

The original contributions presented in the study are included in the article/**Supplementary Material**, further inquiries can be directed to the corresponding author/s.
