# Supplementary material for: Sodium-glucose cotransporter-2 inhibitors in cancer patients with type 2 diabetes and established immune checkpoint inhibitor-related cardiotoxicity: a retrospective analysis
Source: Front Endocrinol (Lausanne). 2026 Apr 15;17:1776717. doi: 10.3389/fendo.2026.1776717 (PMC13126452; doi:10.3389/fendo.2026.1776717)
Supplement: Supplementary file 1 [file Table1.docx]

**Supplementary Table S1. Detailed composition of cancer types in the "Other" category.**

| **Cancer Type** | **Number of Cases (n)** |
| --- | --- |
| Hepatocellular Carcinoma | 5 |
| Pancreatic Cancer | 3 |
| Small Cell Lung Cancer | 3 |
| Colorectal Cancer | 2 |
| Malignant Melanoma | 2 |
| Renal Cell Carcinoma | 2 |
| Diffuse Large B-Cell Lymphoma | 1 |
| Extranodal NK/T-cell Lymphoma | 1 |
| Hodgkin Lymphoma | 1 |
| Hypopharyngeal Squamous Cell Carcinoma | 1 |
| Primary Peritoneal Cancer | 1 |
| Urothelial Carcinoma | 1 |
| Undifferentiated Pleomorphic Sarcoma | 1 |
| Esophageal Small Cell Carcinoma | 1 |
| **Total** | **25** |

This table lists the specific cancer diagnoses that constituted the "Other" category (25.5%) in the primary cohort (N=98), as referenced in the main text, Table 1, and the Results section.
